# Text-to-Speech Screen Reader Accessibility of Phase 3 Consent Documents on ClinicalTrials.gov

**DOI:** 10.1001/jamanetworkopen.2025.30783

**Published:** 2025-09-08

**Authors:** Alexa Lisenby, Nicholas A. Giordano, Solomon Tagbor, Sydney A. Axson

**Affiliations:** 1 Bloomberg School of Public Health, Johns Hopkins University, Baltimore, Maryland; 2 Nell Hodgson Woodruff School of Nursing, Atlanta, Georgia; 3 Ross and Carol Nese College of Nursing, Pennsylvania State University, University Park; 4 Rock Ethics Institute, Pennsylvania State University, University Park

## Abstract

This cross-sectional study examines screen reader accessibility of consent documents in phase 3 trials conducted between 2013 and 2023 and reported on ClinicalTrials.gov.

## Introduction

To promote participation in clinical trials from diverse patient populations, investigators must mitigate barriers to inclusive engagement.^1^ Enhancing informed consent document accessibility for individuals who rely on assistive devices, including approximately 8 million visually impaired people in the US, is essential in this effort.^2^ This analysis examined screen reader accessibility of consent documents in phase 3 trials conducted in the past 10 years and reported on ClinicalTrials.gov.

## Methods

This cross-sectional study used a sample derived from a repository of all National Institutes of Health-funded trials completed between 2013 and 2023 from ClinicalTrials.gov.^3^ Only phase 3 trials with consent documents were included. Phase 3 trials were selected because they often seek to enroll participants with the conditions being investigated.^4^ Consent documents were extracted from ClinicalTrials.gov and read using the Non-Visual Desktop Access (NVDA) screen reader version 2024.1 (NV Access), a free and commonly used screen reader. Characteristics detracting from accessibility, such as headers and footers, images, tables, and flowcharts, were identified to create a Research Electronic Data Capture (REDCap) (version 14.0; Project REDCap) rubric to assess each form. Documents were coded as readable if NVDA could read any part of the document and as unreadable if the NVDA did not identify any words or was incoherent. Classifying a form as readable did not mean there were no issues with the document, but rather that text could be read at any point. One team member (A.L.) coded all documents, and another (S.T.) audited a random 20% of the sample. This analysis did not require institutional review board approval or informed consent, because no individual patient data were used, in accordance with 45 CFR §46, and followed STROBE reporting guidelines.

## Results

In the final sample of 105 documents, 17 (16.0%) were unreadable with the screen reader, 60 (57.1%) had barriers to readability, and 28 (26.7%) had no barriers. When examining the 88 documents with some degree of readability, nearly one-half of the documents (40 documents [45.5%]) were embedded in the trial’s protocol document, which can contain hundreds of pages (Table). Most forms included headers and footers (86 documents [97.7%]), and almost one-half (40 documents [45.5%]) included images. Of the documents with images, 4 (10.0%) included alternative text. Forty-eight documents (54.5%) included tables but only 12 (25.0%) conveyed information as intended. Of the tables that were not understandable, 25 (69.4%) were due to the order that boxes were read, 18 (50.0%) were because they relied on visual cues, and 3 (8.3%) because text was read out of order. Flowcharts appeared in 20 documents (22.7%), but only 2 (10.0%) were accessible. More on table accessibility can be found in the Figure.

**Table.** Characteristics of Phase 3 Consent Documents on ClinicalTrials.gov Accessible With Screen Reader

Characteristics of documents	Documents, No. (%) (N = 88)
Combined with protocol document	40 (45.5)
Included header and/or footers	86 (97.7)
Included images	40 (45.5)
Images had alternative text	4 (10.0)
Included tables	48 (54.5)
Tables understandable with screen reader	12 (25.0)
Table not understandable due to order of boxes	25 (69.4)
Table not understandable due to visual cues	18 (50.0)
Table not understandable due to order of text	3 (8.3)
Included flowcharts	20 (22.7)
Flowchart understandable with screen reader	2 (10.0)

**Figure.**
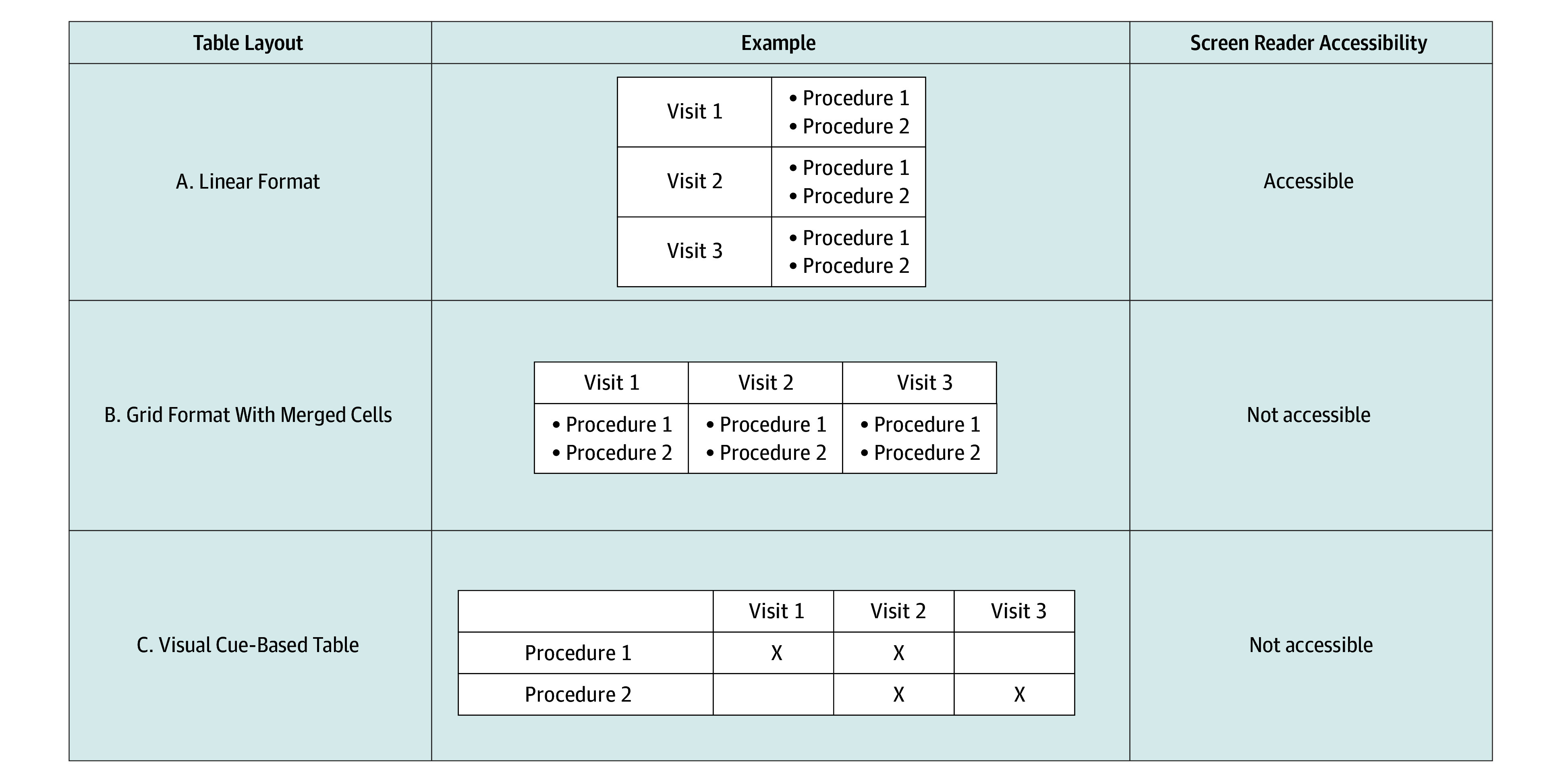
Table Layouts in Informed Consent Forms and Their Screen Reader Accessibility Recommendations provided are based on accessibility determined by 1 screen reader: Non-Visual Desktop Access (NVDA).

## Discussion

This cross-sectional analysis found that among National Institutes of Health–funded phase 3 trials, 16.0% of consent forms were not accessible using a screen reader and the majority of those that were readable contained formatting challenges that hindered accessibility. The use of visuals, tables, and flowcharts has been suggested to make consent forms easier to understand.^5^ We found these elements can present barriers for people using screen readers. Research that is inclusive of persons with visual impairments requires improving consent document accessibility. To enhance accessibility, documents should include descriptive alternative text for images and flowcharts. All information presented in tables should also be written in text. Consent documents on ClinicalTrials.gov should be separate from the trial protocol. Additionally, providing versions without headers and footers will help accessibility. Study limitations include the sample size, use of a single screen reader, lack of input from screen reader users, use of an internally developed rubric, and uncertainty about whether the forms uploaded reflect those used in practice. Despite these limitations, to our knowledge, this analysis is among the first to examine screen reader accessibility of trial consent documents and provides important considerations for posting consent documents on ClinicalTrials.gov in alignment with the revised Common Rule.^6^ Accessible consent forms are an important step toward inclusive clinical trial engagement.

## Notes

Supplement.
Data Sharing Statement
