# Supplementary material for: Text-to-Speech Screen Reader Accessibility of Phase 3 Consent Documents on ClinicalTrials.gov
Source: JAMA Netw Open. 2025 Sep 8;8(9):e2530783. doi: 10.1001/jamanetworkopen.2025.30783 (PMC12418128; doi:10.1001/jamanetworkopen.2025.30783)
Supplement: Supplement. — Data Sharing Statement [file jamanetwopen-e2530783-s001.pdf]

Data Sharing Statement

Data
Data available: No

Additional Information
Explanation for why data not available: The data are already publicly available.
